# Light needle microscopy with spatially transposed detection for axially resolved volumetric imaging

**DOI:** 10.1038/s41598-019-48265-3

**Published:** 2019-08-12

**Authors:** Yuichi Kozawa, Shunichi Sato

**Affiliations:** Institute of Multidisciplinary Research for Advanced Materials, Tohoku University, 2-1-1 Katahira, Aoba-ku, Sendai 980-8577 Japan

**Keywords:** Imaging and sensing, Confocal microscopy

## Abstract

The demand for rapid three-dimensional volumetric imaging is increasing in various fields, including life science. Laser scanning fluorescence microscopy has been widely employed for this purpose; however, a volumetric image is constructed by two-dimensional image stacking with a varying observation plane, ultimately limiting the acquisition speed. Here we propose a method enabling axially resolved volumetric imaging without a moving observation plane in the framework of laser scanning microscopy. A scanning light needle spot with an extended focal depth provides excitation, which normally produces a deep focus image with a loss of depth information. In our method, the depth information is retrieved from transposed lateral information on an array detector by utilising non-diffracting and self-bending characteristics imposed on fluorescent signals. This technique, implemented in two-photon microscopy, achieves truly volumetric images constructed from a single raster scan of a light needle, which has the capability to significantly reduce the acquisition time.

## Introduction

Three-dimensional (3D) volumetric imaging in light microscopy is essential for investigating the detailed structure and functionality of specimens, particularly in life science. Confocal laser scanning microscopy^1^ and multi-photon excitation microscopy^2^ are used for this purpose due to the capability of optical sectioning in the axial direction. Currently, these methods are extensively applied to 3D imaging of biological samples and *in vivo* deep imaging for living samples^3^. In principle, a 3D image can be constructed by stacking multiple two-dimensional (2D) images by changing the observation plane (*z* plane). Thus, the construction of 3D images generally becomes more time-consuming as the depth range increases.

To address this constraint in 3D imaging, a number of techniques utilising multi-spot focusing^4,5^, line focusing^6^ and light-sheet illumination^7^ have been developed, considerably accelerating the acquisition of 2D images compared with conventional laser scanning microscopy. However, these techniques still require mechanical motion of the sample or objective lens, for example, by using a piezo actuator, along the axial direction for 3D image formation, which eventually limits the acquisition speed. Faster axial scanning of an excitation spot can be achieved by using an acoustic optofluidic lens^8^; however, pixel-by-pixel high-throughput data processing synchronised with the axial scanning is required. Alternatively, 3D imaging without mechanical motion of the observation *z* plane has also been realised by various approaches^9–15^. While some of these state-of-the-art techniques demonstrate 3D imaging with a speed of several tens^11,15^ to a hundred^6^ volumes per second, challenges remain in the applicability and implementation of specialised setups for practical situations of biological imaging.

The use of a light needle, or a Bessel beam^16^, in laser scanning microscopy provides an extended depth of field (DOF) for acquired images. This approach has recently become recognised as a rapid volumetric acquisition method for two-photon excitation microscopy^17–22^, particularly for *in vivo* functional imaging of neural activity in a living brain^19,21,22^. With this method, the volume structure of thick samples is visualised within the extended DOF, but the light needle scan obtains images projected along the axial direction. Thus, depth information is lost in the recorded images, which can be retrieved only by additional image acquisitions, namely, conventional *z* stacking for the same field of view^19,21^, or post image analysis^22^. The detection of photoacoustic signals is another technique to retrieve depth information by measuring the time-of-flight signals, which enables motionless volumetric imaging^23^. A similar concept to a Bessel beam was also used to extend DOF in a recent implementation of photoacoustic microscopy^24^. However, compared to laser scanning microscopy, one concern of photoacoustic-based techniques is the limited axial resolution due to the ultrasound nature.

Here, we propose a new approach that can realise truly volumetric imaging from a single raster scan of a light needle in two-photon excitation microscopy. The depth information is directly extracted from emitted signals converted into a well-designed light field, such as an Airy beam, with non-diffracting and self-bending properties. By combining light needle scanning with Airy beam conversion for fluorescent emission, 3D image reconstruction is enabled for fluorescent samples including biological cells without the need to move the observation plane or measure the depth position through additional acquisition procedures. Our method has the potential to capture axially resolved images of entire volumes within an extended DOF at a frame rate identical to that of 2D raster scanning in conventional laser microscopy.

## Results

### Depth retrieving in light needle scanning

**Figure 1:**
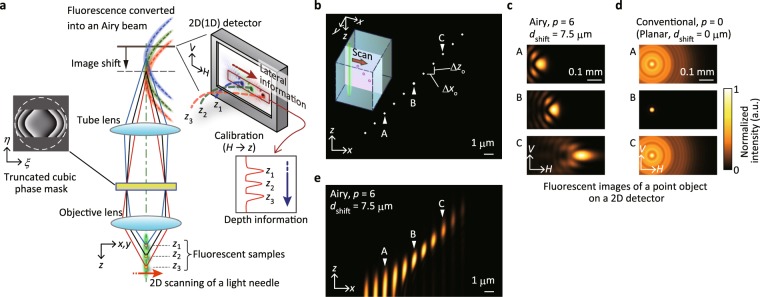
Retrieving depth information from a light needle scan. (**a**) Conceptual diagram. (**b**) Numerical simulation for depth retrieval. Small fluorescent objects (indicated as white dots), arranged on the *xz* plane at the focus with spacings of *Δx*_o_ = *Δz*_o_ = 1 μm, are excited by a light needle that scans along the *x* axis, as illustrated in the inset. (**c**) Calculated fluorescent images of a point object observed at the detector plane (*HV* plane) when the scanning light needle is located at objects A, B and C [as indicated by arrows in (**b**), where *z* = −3, 0 and +3 μm] for *p* = 6 and *d*_shift_ = 7.5 μm. (**d**) Corresponding images observed under conventional (planar) conditions (*p* = 0 and *d*_shift_ = 0). (**e**) Depth calibrated *xz* plane image of point objects acquired for *p* = 6 with *d*_shift_ = 7.5 μm.

A schematic of our method is shown in Fig. 1a. Our method relies on 2D scanning of a light needle with an extended DOF, readily realised by focusing an annular-shaped beam or using an Axicon lens^16^. At each scanning position, fluorophores of a sample located within the light needle are simultaneously excited and emit fluorescent signals. Normally, detection of all of the signals using a point detector, such as a photo-multiplier tube (PMT), merely produces a deep focus image^25,26^, for which the depth information is lost.

To retrieve the depth information from fluorescent emission, we utilise the unique features of an Airy beam^27^, for which the centroid laterally shifts along a parabolic trajectory without diffraction spreading during propagation. Over the past decade, these properties of Airy beams have been applied in a variety of fields including optical manipulation^28^, light-sheet microscopy^12,29^, plasma-channel generation^30^, laser processing^31^ and 3D super-resolution imaging^32^. We apply the non-diffracting and self-bending characteristics to fluorescent emission by imposing a cubic phase modulation^27^—along with a rectangular mask^32^ and a Fresnel lens—at the pupil plane of an objective lens. This spatial phase modulation generates an Airy beam characterised by a parameter *p* as well as the axial displacement of the image plane represented by *d*_shift_, which is needed to ensure depth extraction (see Methods). Then, an image converted into an Airy beam is formed at a different lateral position (along the *H* axis) on the image plane (*HV* plane) corresponding to the *z* position in the object space (see Fig. 1a). Consequently, the intensity profile measured along the *H* axis reflects the spatial distribution of fluorophores (*z*_1_, *z*_2_ and *z*_3_) along the *z* axis of the specimen. By calibrating the relation between the *z* and *H* axes, we can directly obtain the depth information of objects from the transposed lateral information in the image plane by using an array detector.

Based on this concept, numerical simulations were performed to examine the capability of depth extraction. As an observation sample, we considered an array of infinitely small fluorescent objects aligned in the *xz* plane in the object space (Fig. 1b). These objects are excited by a light needle that scans along the *x* axis in the *xz* plane. The light needle considered here is assumed to have an infinitely extended DOF and a lateral spot size equal to that obtained by focusing a conventional circularly polarised beam. In our simulation, we used numerical conditions similar to those employed in our experiments, as described later.

Figure 1c displays the calculated fluorescent images on the detector when the light needle illuminates the point objects indicated as A, B and C in Fig. 1b. We assumed *p* = 6 and *d*_shift_ = 7.5 μm for Airy beam conversion by considering a sufficient axial separation within the detectable depth range (see Methods and Supplementary Fig. S1). In the detector plane, the image for each point object shows a characteristic intensity distribution following an Airy beam pattern with reduced side lobes. Note that residual side lobes still remain around the centre main lobe in the detector plane. Nevertheless, these side lobes have a negligible impact on the reconstructed image quality because only the intensity profile along the *H* axis across the centre main lobe is used to reconstruct the axial information. Importantly, the converted images laterally shift along the *H* axis in accordance with the *z* position of the point objects (*z* = −3, 0 and 3 μm) owing to the self-bending property of the Airy beam. In contrast, the case without phase modulation (*p* = 0 and *d*_shift_ = 0, see Fig. 1d) results in spatially blurred, defocused images, except for the object at *z* = 0. This numerical simulation also illustrates the non-linear relation between the lateral shift in the image space and the depth position in the object space due to the parabolic trajectory of the Airy beam propagation. To correct this non-linearity, the *H* axis was calibrated regarding the *z* axis by evaluating the centre position of each image converted into an Airy beam (see Methods).

With the acquired calibration curve, we can reconstruct the depth profile directly from the intensity profile along the *H* axis by assigning the pixels on the *H* axis to corresponding *z* positions. Figure 1e shows a reconstructed *xz* image for the point objects (Fig. 1b), which clearly demonstrates that the depth of the point objects is retrieved. The reconstructed *xz* image also shows that the images of the point objects in the lower region are elongated along the *z* axis, where the elongation decreases as *z* increases. This trend implies that the axial resolution depends on the *z* position due to the parabolic nature of Airy beam propagation. By thoroughly analysing the numerical simulations, the size of a point object image, or the point spread function (PSF), was evaluated for our approach (see Supplementary Fig. S2). The full-width at half-maximum (FWHM) value of an axial PSF for *p* = 6 and *d*_shift_ = 7.5 μm varies in the range of 0.92–2.98 μm within the depth range of *z* = −3.5 to 4.5 μm. By contrast, the lateral PSF size within the same depth range is approximately 0.32 μm on average and varies slightly with *z*. For comparison, in conventional two-photon excitation microscopy, the axial and lateral PSF sizes are 1.0 and 0.36 μm, respectively, when a circularly polarised plane wave is assumed for the same conditions. This evaluation reveals that our method offers an axial spatial resolution on the order of that obtained in conventional two-photon excitation microscopy. Interestingly, an increased spatial resolution in both the axial and lateral directions is also expected for a limited depth range. This resolution improvement is attributed to an effect equivalent to that of a confocal pinhole^33^, because the detected signal assigned to each pixel in the reconstructed image is spatially restricted within a small region on the detector plane.

### Experimental demonstration

To demonstrate 3D image reconstruction, we developed a laser scanning microscope system that employs light needle scanning for excitation and Airy beam conversion for detection (see Methods and Supplementary Fig. S3 for system details). A femtosecond pulsed laser source with a wavelength of 1040 nm for two-photon excitation was converted into a thin-annular-shaped beam by using a spatial light modulator (SLM). The annular-shaped beam generates a needle-shaped Bessel beam at the focus of a water immersion objective lens with a numerical aperture (NA) of 1.15. The width and diameter of the annular shape were designed to produce a light needle with a 15-fold DOF and a similar lateral spot size compared with conventional beam focusing.

**Figure 2:**
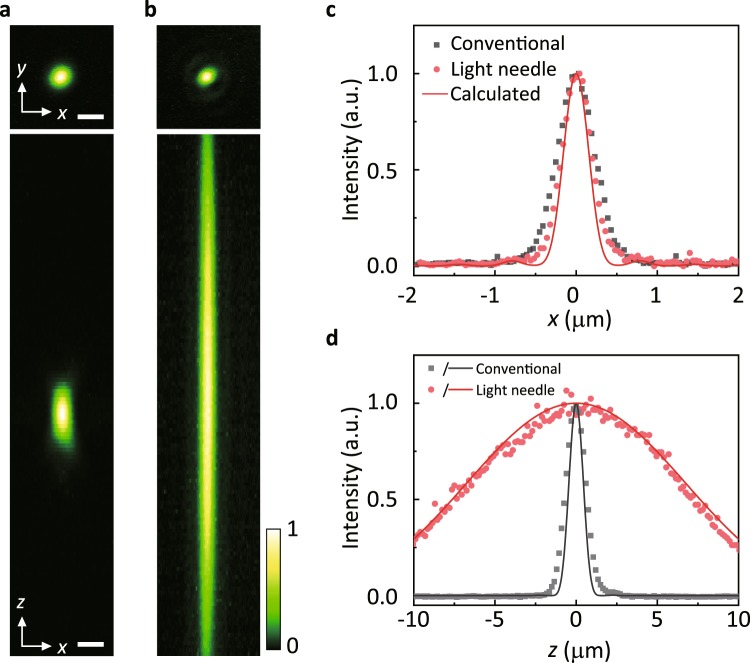
Two-photon excitation light needle with an extended DOF. (**a**,**b**) Measured intensity distributions of focused excitation beams in the *xy* (top) and *xz* planes (bottom) for conventional conditions (**a**) and light needle focusing (**b**) designed to realise a 15-fold DOF compared to conventional focusing. The scale bars in (**a**) are 1 μm. (**c**,**d**) Measured intensity profiles (filled circles and squares) and simulated results (solid lines) along the *x* (**c**) and *z* (**d**) axes.

Compared with the measured two-photon excitation PSF for conventional beam focusing (Fig. 2a), the generated light needle PSF has a sufficiently extended DOF (Fig. 2b), as expected (see Fig. 2c,d). The FWHM values of the light needle PSF measured along the *z* and *x* axes are 14.6 and 0.43 μm, respectively, which are comparable to the designed values (15 and 0.36 μm). The measured light needle has no apparent side lobes around the centre spot, whereas focusing of a thin-annular-shaped beam is known to produce numerous side lobes at the focus based on its nature as a Bessel beam. This side lobe reduction is due to the two-photon excitation process, in which the fluorescence excitation probability is proportional to the square of the focal intensity^34^. Consequently, the light needle enables excitation only along the depth direction without undesirable side lobes, with a range of more than 10 μm at each scanning position.

To examine the lateral shifting behaviour of Airy beam images, we evaluated fluorescent images of a point object using our setup. An isolated orange bead with a diameter of 200 nm was placed on the light needle and moved along the *z* axis by a piezo stage. At each *z* position, we recorded the emitted fluorescent signal using an electron-multiplying charge-coupled device (EMCCD) camera through a detection path after applying a cubic phase modulation using another SLM.

**Figure 3:**
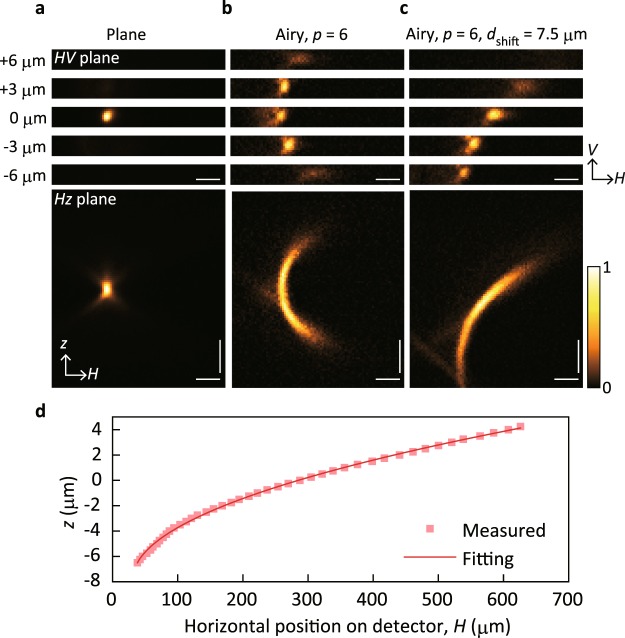
Fluorescent images with Airy beam conversion for depth calibration. (**a**–**c**) Fluorescent images (measured on the detector plane, *HV* plane) of an isolated orange bead without any phase modulation (**a**) and with phase modulation with *p* = 6 only (**b**) and *p* = 6 and *d*_shift_ = 7.5 μm (**c**). The top five panels present images recorded when the bead was located at *z* = −6 μm to 6 μm with an axial pitch of 3 μm. The bottom panels in (**a**–**c**) show images on the reconstructed *Hz* plane with an extended moving range. The horizontal and vertical scale bars are 200 and 5 μm, respectively. (**d**) Relationship between the horizontal position on the image plane and the axial position in the object space evaluated from the bottom panel in (**c**) and the fitting result obtained using a square root function (see Methods).

For the conventional imaging condition, where the fluorescent signal was focused on the image plane without any phase modulation (*p* = 0 and *d*_shift_ = 0), the bead image was observed only when *z* = 0, as shown in Fig. 3a. For the cubic phase modulation (*p* = 6, see Fig. 3b,c), the fluorescent signal was detected as an image converted into an Airy beam in the extended depth range. The recorded images clearly demonstrate a depth-dependent lateral shift along the *H* axis, reflecting the self-bending nature of Airy beam propagation. However, without the axial displacement (*d*_shift_ = 0, Fig. 3b), two images are indistinguishable on the detector when the bead is located before or after the focus with the same distance from *z* = 0. We avoided this situation by superimposing a Fresnel lens pattern on the SLM to displace the image plane by a distance corresponding to *d*_shift_ = 7.5 μm, as shown in Fig. 3c. The centre position of the bead image was monotonically (but non-linearly) shifted along the *H* axis, depending on *z*. We then obtained the calibration curve plotted in Fig. 3d to correct for the non-linearity by curve fitting with a square root function, which was evaluated for the centre positions of the bead images as a function of *z* (see Methods). Similar calibration curve was used in a subsequent experiment to reconstruct 3D images.

**Figure 4:**
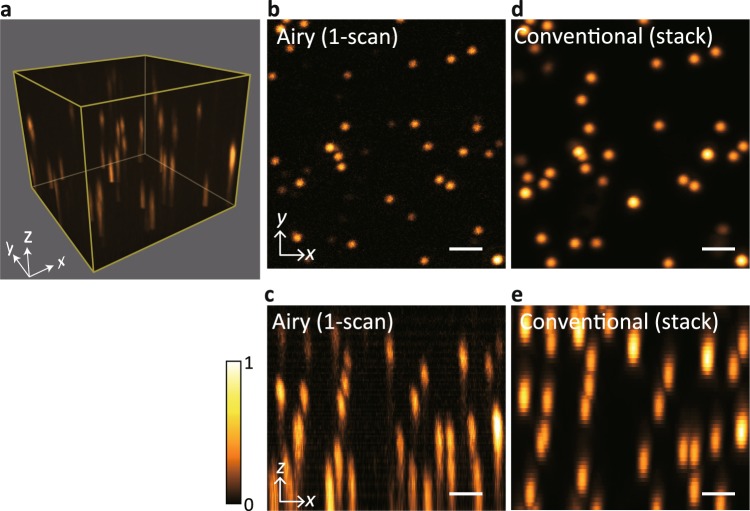
Reconstruction of 3D images for fluorescent beads. (**a**) Reconstructed 3D image of fluorescent beads embedded in agarose gel using light needle scanning with Airy beam conversion (*p* = 6, *d*_shift_ = 7.5 μm). The rendered volume size is 14.5 × 14.8 × 12.2 μm^3^. (**b**,**c**) Maximum intensity projection (MIP) for the *xy* and *xz* planes produced from (**a**). (**d**,**e**) Corresponding MIP images obtained by conventional 50-image stacking. The scale bar in each panel is 2 μm.

Our method was applied to image 200-nm-diameter orange fluorescent beads embedded in agarose gel. We recorded the fluorescent images using the EMCCD in synchronisation with raster scanning of the light needle. The series of acquired images was then processed to reconstruct a 3D volumetric image, as shown in Fig. 4a–c. For comparison, the same field of view was imaged through conventional image stacking, using 50 images to produce a 3D image (Fig. 4d,e). By comparing these results, we verified that the depth position of each bead within the reconstructed volume (14.5 × 14.8 × 12.2 μm^3^) was successfully retrieved by our method. Thus, the present result proves the capability of 3D image reconstruction from a single 2D scan of the light needle without a change in the observation plane. Figure 4c also presents the depth-dependent variation of the axial resolution, as mentioned in the preceding section. We evaluated the axial and lateral resolutions by measuring bead sizes observed at several *z* planes (see Supplementary Fig. S5), which follow a tendency similar to that predicted in the numerical simulations.

**Figure 5:**
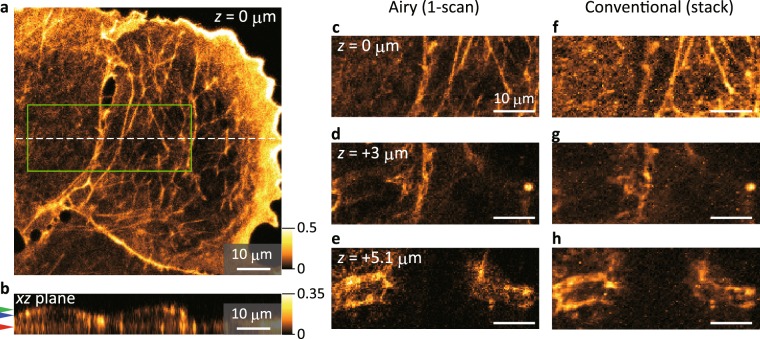
Imaging for biological cells. (**a**) Reconstructed *xy* image (at *z* = 0 μm) for F-actin in fixed COS-7 cells stained by Alexa Fluor 532. The images were captured by light needle scanning with Airy beam conversion (*p* = 6, *d*_shift_ = 7.5 μm). The acquired and reconstructed volume size is 80 × 80 × 14.7 μm^3^. (**b**) Cross-sectional *xz* image across the white dashed line in (**a**). (**b**,**c**) Magnified images of the rectangular region indicated in (**a**), extracted from the reconstructed 3D image for *z* = 0 μm (**c**), 3 μm (**d**) and 5.1 μm (**e**), whose *z* planes are also indicated by red, blue and green arrows, respectively, in (**b**). (**f**–**h**) The corresponding images of the same region acquired by conventional laser scanning microscopy by changing the observation plane. The intensity of the images shown in (**a**) and (**b**) is oversaturated with respect to the maximum intensity (normalised to 1) for each image to visualise the weak signals, as indicated in the colour scales. The scale bars shown in (**c**–**h**) are 10 μm.

We further examined the applicability of our method for biological samples. The present setup enables 3D visualisation of samples within a depth range of approximately 10 μm with axial and lateral spatial resolutions comparable to those of conventional two-photon microscopy. This result suggests that volumetric imaging of an entire biological cell can be accomplished in a single raster scan. We acquired and reconstructed a 3D image of F-actin structures in fixed COS-7 cells labelled with phalloidin-conjugated Alexa Fluor 532, as shown in Fig. 5a and Supplementary Video 1. The entire depth profile of the cells was well visualised (see Fig. 5b), demonstrating the optical sectioning ability of this approach (see Fig. 5c–e). The same volume was acquired by conventional image stacking with a focused Gaussian beam as shown in Fig. 5f–h. The images obtained for each axial position are comparable to those reconstructed by our method, which supports the imaging capability of the present approach even for samples with complicated structures such as biological samples. The present results were obtained by light needle scanning with a laser power of 42 mW at the focus, whereas a Gaussian beam with a power of a few milliwatts was generally used to produce corresponding conventional images in our setup. Nonetheless, no obvious photo-bleaching was observed during imaging. As one reason for this finding, the annular beam focusing in the present setup produces a focal spot with a peak intensity equal to ~7% of that produced by conventional beam focusing with the same laser power. Most of the focused laser energy is spread around the focus as weak side lobes, which contribute little to the two-photon excitation for fluorophores. This feature can also be viewed from another aspect: our method has a significant advantage in preventing photo-bleaching because repeated raster scans are unnecessary to construct 3D images.

## Discussion

In the present setup, the total acquisition speed is limited primarily by the frame rate of the EMCCD camera. Even capturing fluorescent signals by the EMCCD camera at several thousand of frames per second corresponds to a pixel dwell time on the order of one hundred microseconds in our method. Compared to conventional laser scanning microscopy, this process is slow. However, the limitation of the acquisition speed can be overcome by simply employing parallel highly sensitive photo detectors such as a PMT array^35^ or a single-photon avalanche diode array^36^, as implemented in image scanning microscopy. Ultimately, our method requires only a one-dimensional detector array aligned along the *H* axis to retrieve the depth information. The utilisation of these advanced technologies as well as faster laser scanning using a resonant scanner^21^ will further increase the acquisition speed for volumetric imaging.

In our method, the detectable depth range and axial resolution depend primarily on the non-diffracting and self-bending characteristics of an Airy beam. An Airy beam with a larger *p* provides a greater non-diffraction length but reduces the rate of lateral shifting regarding propagation^27,30^. This feature results in a trade-off between the detectable depth range and the axial spatial resolution, which can be controlled by the applied *p* value in phase modulation. Additionally, a larger *p* value decreases the centre peak intensity of an Airy beam, reducing the signal intensity. Thus, an appropriate *p* value should be chosen by considering the required depth range and spatial resolution as well as the sensitivity of the detector.

To improve the depth range and spatial resolution in 3D reconstruction, different approaches without Airy beam conversion can also be applied. In our method, ideal behaviour can be imposed on the fluorescence emission if the converted PSF exhibits a non-diffracting and linear-shifting behaviour along the *H* axis with respect to *z*. The use of a computer-generated hologram (CGH) that directly tailors the PSF behaviour is a promising route for this purpose. Recently, we have demonstrated a non-diffracting and linear-shifting PSF using a properly designed CGH mask in a bench-top experiment^37^. The implementation of such engineered PSFs based on the CGH technique, combined with an adequate conversion efficiency under a fluorescent microscope setup, will enable 3D reconstruction for a greater depth range from a simple linear relationship between the *H* and *z* axes.

In conclusion, we have proposed a novel method for retrieving depth information in light needle scanning microscopy by imposing non-diffracting and self-bending properties on fluorescent signals. Our method enables 3D image reconstruction from a single 2D raster scan of a light needle, potentially increasing the acquisition speed for volumetric imaging compared to conventional image stacking. The reconstruction of 3D images for fluorescent samples, including biological samples, was demonstrated in the framework of conventional laser scanning microscopy. Thus, this technique can be applied to a variety of practical situations in imaging, for example, particle tracking, flow cytometry, visualisation of the dynamical motion of entire living cells and *in vivo* functional imaging.

## Methods

### Pupil modulation for Airy beam conversion

Considering the inherent nature of an Airy beam, we must carefully consider the following aspects to correctly extract the depth information. One aspect is the presence of side lobes around the centre main lobe of an Airy beam. These side lobes overlap at the image plane, causing degradation and artefacts in the reconstructed axial profile. However, such undesired side lobes can be effectively removed by an appropriately designed rectangular mask with a cubic phase modulation, as reported by Jia *et al*.^32^. Another aspect is the absence of a unique solution for depth extraction due to the symmetrical parabolic trajectory in Airy beam propagation. Specifically, two objects located at the same distance before and after the focus cannot be distinguished. To avoid this indeterministic situation, in our method, we superimposed a Fresnel lens pattern on the pupil modulation function to shift the image plane along the axial direction. This approach ensures that the depth extraction is performed using half of the parabolic trajectory in Airy beam propagation (see Supplementary Fig. S1).

It is assumed that the Airy beam conversion is achieved by using, for example, a phase-only SLM at the pupil plane of the objective lens. The applied phase function is expressed as

1 $$W(\xi ,\eta )={W}_{{\rm{cubic}}}(\xi ,\eta )+{W}_{{\rm{lens}}}(\xi ,\eta )$$

where (*ξ*, *η*) are the coordinates in the pupil plane for a radius *a*.

${W}_{{\rm{cubic}}}(\xi ,\eta )=2{\rm{\pi }}p[{(\xi +\eta )}^{3}+{(\xi -\eta )}^{3}]/(2\sqrt{2}{a}^{3})$ denotes a cubic phase function, where *p* is a parameter that determines the non-diffraction length and the lateral shifting behaviour of a converted Airy beam. ${W}_{{\rm{l}}{\rm{e}}{\rm{n}}{\rm{s}}}(\xi ,\eta )=-k({\xi }^{2}+{\eta }^{2})/(2{f}_{SLM})$ represents a Fresnel lens, where *f*_SLM_ is the focal length and *k* is the wavenumber of the fluorescent emission. The applied Fresnel lens displaces the image plane in the axial direction (toward the tube lens for *f*_SLM_ > 0) with a distance ${D}_{{\rm{shift}}}={f}_{{\rm{t}}}^{2}/{f}_{{\rm{SLM}}}$, where *f*_t_ is the focal length of the tube lens. The axial image shift *D*_shift_ in the image space can also be measured by the distance *d*_shift_ scaled in the object space from the relation ${d}_{{\rm{shift}}}={D}_{{\rm{shift}}}/{M}_{{\rm{L}}}$, where *M*_L_ is the longitudinal magnification of the imaging optics. In the present study, for convenience, we refer to the extent of the axial image shift as the corresponding displacement *d*_shift_. The amplitude of the spatially modulated fluorescent emission is then truncated by a rectangular aperture, using a rectangular function expressed as ${\rm{rect}}(\frac{\eta }{2ah})$, where *h* represents the slit size and is appropriately chosen to reduce the side lobes of the Airy beam images^32^. Finally, the converted fluorescent emission is captured as an image by an array detector placed at the focal plane of the tube lens.

### Image formation of fluorescent emission with Airy beam conversion

We consider the image of a fluorescent point object through imaging optics after the electric field of the fluorescent emission is modulated at the pupil plane of a high-NA objective lens. For simplicity, we omit any polarisation effects in fluorescent emission and detection, which are normally considered in high-NA conditions^38^. Thus, a point object is assumed to emit a spherical wave with random polarisation as fluorescent emission. First, we assume a point object located on the optical axis at (0, 0, *z*_o_). Let *E*_em_ be the scalar electric field of an emitted fluorescent signal at the pupil plane of the objective lens. The fluorescent signal has a defocused wavefront in accordance with the axial position *z*_o_, which is expressed as

2 $${E}_{{\rm{e}}{\rm{m}}}(\xi ,\eta )={E}_{0}\,\exp [ink{z}_{{\rm{o}}}\sqrt{1-\frac{N{A}^{2}}{{a}^{2}{n}^{2}}({\xi }^{2}+{\eta }^{2})}]$$

where *E*_0_ is the amplitude of the electric field and the exponential term corresponds to a defocused wavefront derived from a rigorous consideration under high-NA conditions^39,40^, with *n* being the refractive index of the medium. To impose self-bending behaviour, *E*_em_ is modulated by a SLM with a phase function and an amplitude mask using Eq. (1) as

3 $${E}_{{\rm{pupil}}}(\xi ,\eta )={E}_{{\rm{em}}}(\xi ,\eta )\exp [iW(\xi ,\eta )]{\rm{rect}}(\frac{\eta }{2ah})$$

In our setup, a tube lens is placed at a distance *f*_t_ from the pupil plane, which guarantees a Fourier transform relationship between the pupil plane and the image plane. Consequently, the intensity distribution of the fluorescent image at the image plane (detector plane) is written as ${I}_{{\rm{p}},0}(H,V)={| {\mathcal F} ({E}_{{\rm{pupil}}})|}^{2}$, where (*H*, *V*) are the coordinates in the image plane and ${\mathcal F}$ denotes a Fourier transform. Then, by using a simple imaging relation, we suppose that a point object located at (*x*_o_, *y*_o_, *z*_o_) produces an image expressed as ${I}_{{\rm{p}}}({\rm{H}},{\rm{V}})={I}_{{\rm{p}},0}(H+M{x}_{0},V+M{y}_{0})$, where *M* is the lateral magnification of the system. Finally, when the scanning light needle is positioned at (*x*_s_, *y*_s_, 0) in the object space, the detected signal for the point object is written as ${I}_{{\rm{\det }}}(H,V;{x}_{{\rm{s}}},{y}_{s})={I}_{{\rm{p}}}(H,V)\cdot {{\rm{PSF}}}_{{\rm{needle}}}({x}_{{\rm{s}}}-{x}_{{\rm{o}}},{y}_{{\rm{s}}}-{y}_{{\rm{o}}},{z}_{{\rm{o}}})$, where ${{\rm{PSF}}}_{{\rm{needle}}}(x,y,z)$ represents the two-photon excitation PSF obtained by the square of the focal spot corresponding to the light needle. The above consideration is applicable to general fluorescent objects comprised of many point objects, given that fluorescent emission can be incoherently superposed at the image plane. The resultant image is obtained by the superposition of the *I*_det_ produced by each point object.

In our numerical simulation, we assumed that a laser beam with a wavelength of 1040 nm, used as a two-photon excitation beam, is focused by a water immersion objective lens with NA = 1.15 and *n* = 1.33. Fluorescent emission with a wavelength of 560 nm at each scanning position is collected by the objective lens and then focused by a tube lens (*f*_t_ = 400 mm) after phase modulation to produce an Airy beam image with a magnification *M* = 80. PSF_needle_ was calculated using vector diffraction theory^41^ by considering the focusing of an annularly masked, circularly polarised beam. The inner and outer radii of the annular mask were chosen to be 0.674*a* and 0.724*a* with pupil radius *a* = 5.75 mm to produce a PSF_needle_ with a 15-fold DOF compared with the conventional PSF with plane wave focusing. In this condition, the lateral spot size of the PSF_needle_ is similar to that of the conventional PSF. Note that in the numerical simulation, we considered only the lateral intensity profile of the PSF_needle_, namely, ${{\rm{PSF}}}_{{\rm{needle}}}(x,y,z)={{\rm{PSF}}}_{{\rm{needle}}}(x,y,0)$, to clarify the behaviour of Airy beam images. The slit size parameter *h* in Eq. (3) for Airy beam conversion was set to 0.48*a*, 0.41*a* and 0.37*a* for *p* = 4, 6 and 8, respectively, to reduce the intensity of side lobes in the resultant image^32^. These parameters were also adopted in the experimental study.

The results of numerical simulations for the image formation of point objects aligned in the *xz* plane are summarised in Supplementary Fig. S1, which exhibits the role of *p* and *d*_shift_ in the image formation with Airy beam conversion. The point image with Airy beam conversion forms at different position on the *H* axis with respect to the *z* position of the object in the *xz* plane, reflecting the self-bending behavior of an Airy beam. This position shift caused by the self-bending behavior is reduced with increasing the value of *p*, whereas the depth-invariant image formation by the non-diffracting behavior is enhanced. Additionally, Airy beam conversion with a larger *p* value reduces the signal intensity of the converted point images. Fig. S1(c) also displays that a unique solution of the depth extraction is obtained when *d*_shift_ > 0. In principle, *d*_shift_ should be larger than half of the depth range to be extracted. Owing to the parabolic trajectory of Airy beam propagation, larger *d*_shift_ is favorable to obtain sufficient lateral shift within the observed depth range, which increases the axial spatial resolution in reconstructed images. By considering these features, we used *p* = 6 and *d*_shift_ = 7.5 μm in both numerical simulation and experiment.

### Calibration for depth extraction

To calibrate the relation between the *H* and *z* axes, we obtained images of an isolated point object moving along a light needle. For each *z* position of the point object, the peak position on the *H* axis across the centre of the point image was determined by curve fitting with a Gaussian function. In the numerical simulations, we calibrated the *H* axis to directly assign the *z* axis by linear interpolation from the collected values of the evaluated pair (*z*, *H*). In the experiments, the *z* position calibrated regarding the measured centre position *H* was approximated by a square root function expressed as $z={z}_{0}+B\sqrt{H-{H}_{0}}$, where *z*_0_, *H*_0_ and *B* are parameters determined by curve fitting to the evaluated position data. In our experiments, calibration was performed within the depth range that provides a peak intensity (signal intensity) larger than 1/e^2^ of the maximum intensity measured within the DOF of the light needle.

### Experimental setup

A femtosecond pulsed laser source (femtoTRAIN 1040-5, Spectra-Physics) with a wavelength of 1040 nm, a repetition rate of 10 MHz and a pulse width below 200 fs was used for two-photon excitation in our setup. A light needle was generated for the excitation beam by utilising a phase-only SLM (SLM-100, Santec) placed in the excitation light path. The SLM imposed an annular-shaped phase mask on the incident beam by displaying a linear phase grating within the annular region to apply a wavefront tilt with an angle of 0.5 degrees in the vertical direction. Only the first-order diffraction light was filtered out by an aperture placed in the Fourier plane. The generated annular beam was projected on the pupil plane of a water immersion objective lens (CFI Apochromat LWD Lambda S 40XC WI, Nikon) with NA = 1.15 using relay optics; the beam was then focused to form a light-needle-shaped Bessel beam at the focus. Additionally, before focusing, the annular beam was converted into a circularly polarized beam by a quarter waveplate. The focused excitation beam was raster-scanned using Galvano scanning mirrors (8315KM40B, Cambridge Technology). The observation sample was mounted on a piezo stage (Nano-PDQ350HS, Mad City Labs) to precisely control the sample position for calibration and image stacking in conventional 3D imaging. An emitted fluorescent signal was separated by a dichroic mirror (NFD01-1040-25 × 36, Semrock) with an additional notch filter (#86-128, Edmund Optics) and projected to another SLM (SLM-100, Santec) in the detection path to apply a truncated cubic phase function. We assumed that fluorescent signal is at a centre wavelength of 560 nm. Experimental values of the parameters used for the annular mask and the truncated cubic phase function are given in the preceding sections. Here, for Airy beam conversion, the discrete nature of pixelated SLM may need to be considered if an applied phase has a steep variation beyond the spatial resolution of the SLM. The SLM used in our experiments has a pixel pitch of 10.4 μm with a pixel size of 10 μm. In most cases, we adopted the experimental condition of *p* = 6 and *d*_shift_ = 7.5 μm for Airy beam conversion, which has the steepest phase variation of 1*λ*, where *λ* = 560 nm, within 0.17 mm corresponding to 16 pixels in the SLM (see Supplementary Fig. S4). Thus, the phase modulation adopted in our experiments can be correctly applied to the fluorescent signals. The signal converted into an Airy beam was then focused on an EMCCD camera (iXon Ultra 897, Andor). Image acquisition was performed by raster scanning of a light needle synchronised to continuous image capturing in the EMCCD camera with a frame rate of 2000 (per second), corresponding to a pixel dwell time of 0.5 ms for raster scanning. For conventional two-photon imaging, the fluorescent signal was detected through another detection path equipped with a photodetector (R10467U-40, Hamamatsu). No pinhole was used in the signal detection. Supplementary Fig. S3 shows the detailed experimental setup.

Notably, in our setup, the phase modulation for fluorescent signals relies on a polarisation-dependent, liquid-crystal-based SLM. Thus, half of the fluorescent emission with random polarisation cannot be used for image reconstruction, reducing the resultant signal level. The use of a polarisation-independent deformable mirror is a promising alternative for improving the signal level, which can convert all of the emitted fluorescence into an Airy beam.

### Sample preparation

Fluorescent orange beads with a diameter of 200 nm (FluoSpheres, Invitrogen) suspended in water were dropped on a coverslip and dried. The inverted coverslip was placed on a glass slide with a small amount of water and sealed with nail polish. The prepared slide was used for the PSF measurement and the calibration process. For the 3D imaging demonstration, fluorescent beads sparsely distributed in agarose gel were used. For this purpose, the same 200-nm beads were embedded in agarose gel (1% agarose L, Nippon Gene), which was mounted on a glass slide using a slide seal and covered by a coverslip.

COS-7 cells (DS Pharma Biomedical) were cultured on a coverslip with Dulbecco’s modified Eagle’s medium (Fujifilm Wako Pure Chemical) supplemented with 10% foetal bovine serum in a CO_2_ incubator at 37 °C. The cells were then fixed with 4% paraformaldehyde and stained with Alexa Fluor 532 phalloidin (A22282, Invitrogen). The stained cells were mounted on a glass slide with an antifade reagent (SlowFade Diamond Antifade, Invitrogen).

## Supplementary information

Reconstructed 3D image of biological cells

Supplementary information

## Data Availability

The datasets generated during and/or analysed during the current study are available from the corresponding author on reasonable request.
