# Supplementary material for: Light needle microscopy with spatially transposed detection for axially resolved volumetric imaging
Source: Sci Rep. 2019 Aug 12;9:11687. doi: 10.1038/s41598-019-48265-3 (PMC6690918; doi:10.1038/s41598-019-48265-3)
Supplement: Supplementary file 2 — Supplementary information [file 41598_2019_48265_MOESM2_ESM.pdf]

Supplementary information

# Light needle microscopy with spatially transposed detection for axially resolved volumetric imaging

**Yuichi Kozawa* and Shunichi Sato**

*Institute of Multidisciplinary Research for Advanced Materials, Tohoku University, 2-1-1 Katahira, Aoba-ku, Sendai 980-8577, Japan*

**Corresponding author: y.kozawa@tohoku.ac.jp*

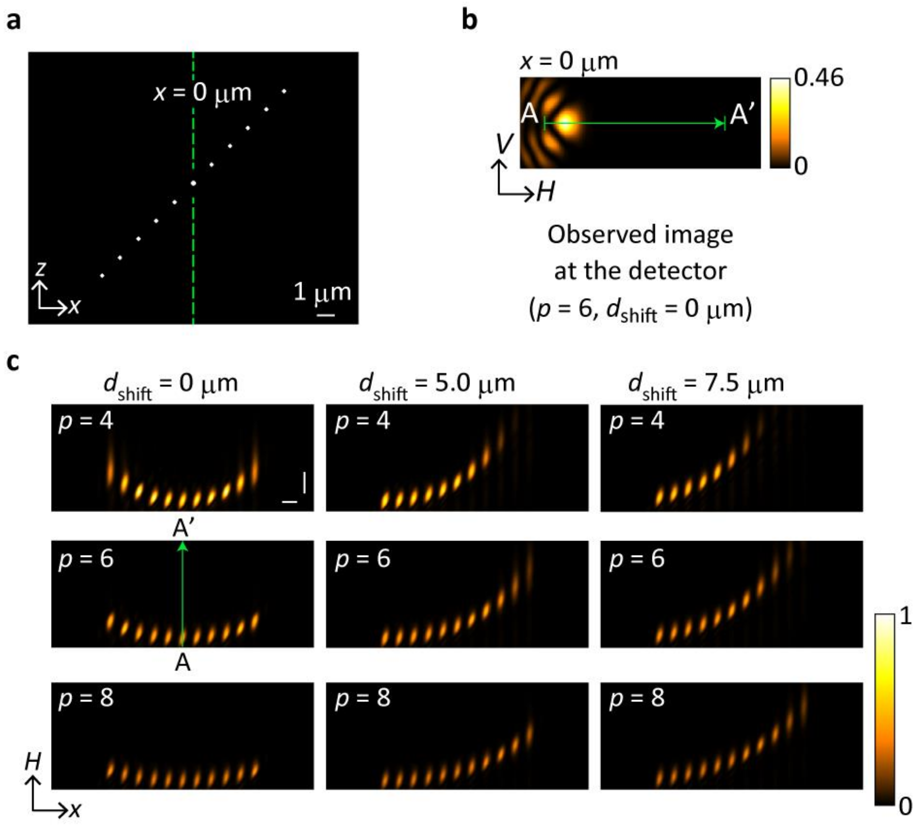


**Supplementary Figure S1.** Numerical simulation of Airy beam images for point objects. (**a**) Examined point objects aligned in the *xz* plane (identical to that shown in Fig. 1b). (**b**) An example of a calculated image at the detector plane (*HV* plane) for $p = 6$ and $d_{\mathrm{shift}} = 0$ μm when the light needle is located at $x = 0$ μm, denoted by the green dashed line in (**a**). (**c**) Constructed *xH* plane images for different $p$ and $d_{\mathrm{shift}}$ values. The intensity profile across the centre of the image along the A-A’ line depicted in (**b**) is utilised as the vertical axis of the constructed *xH* images (see the left middle panel). The colour scale in each panel displayed in (**b**) and (**c**) is normalised to the maximum value for $p = 4$ and $d_{\mathrm{shift}} = 0$ μm (upper left). The horizontal and vertical scale bars are 1 μm and 0.1 mm, respectively.

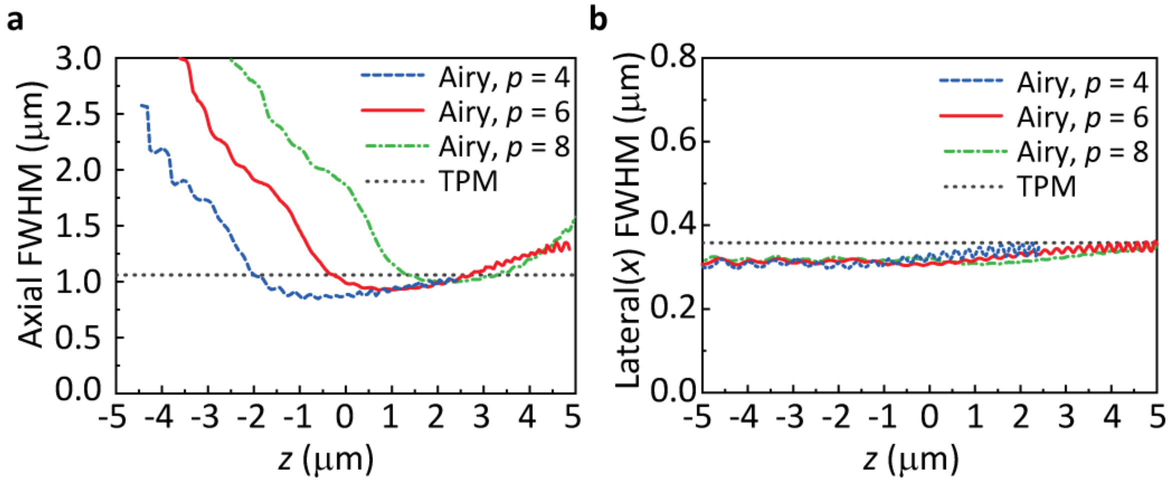


**Supplementary Figure S2.** Evaluation of the spatial resolution for PSFs with Airy beam conversion as a function of the axial position. (**a**) Axial PSF size calculated for $p$ = 4 (blue dashed line), 6 (red solid line) and 8 (green dashed-dotted line). The results for conventional two-photon excitation microscopy are shown by a black dotted line. For Airy beam conversion, an axial image shift of $d_{\text{shift}}$ = 7.5 μm is considered for all cases. (**b**) Corresponding lateral PSF sizes along the $x$ axis. In both figures, the calculated sizes for Airy beam PSFs show a depth-dependent variation with small short-period oscillations. This small oscillation is attributed to diffraction ripples appearing in the converted point images (data not shown), which affect the numerical evaluation of the FWHM size for each resultant image. The PSF size is determined within the depth range for which the peak intensity of each converted point image is greater than 5% of the maximum intensity obtained within a depth range of 10 μm.

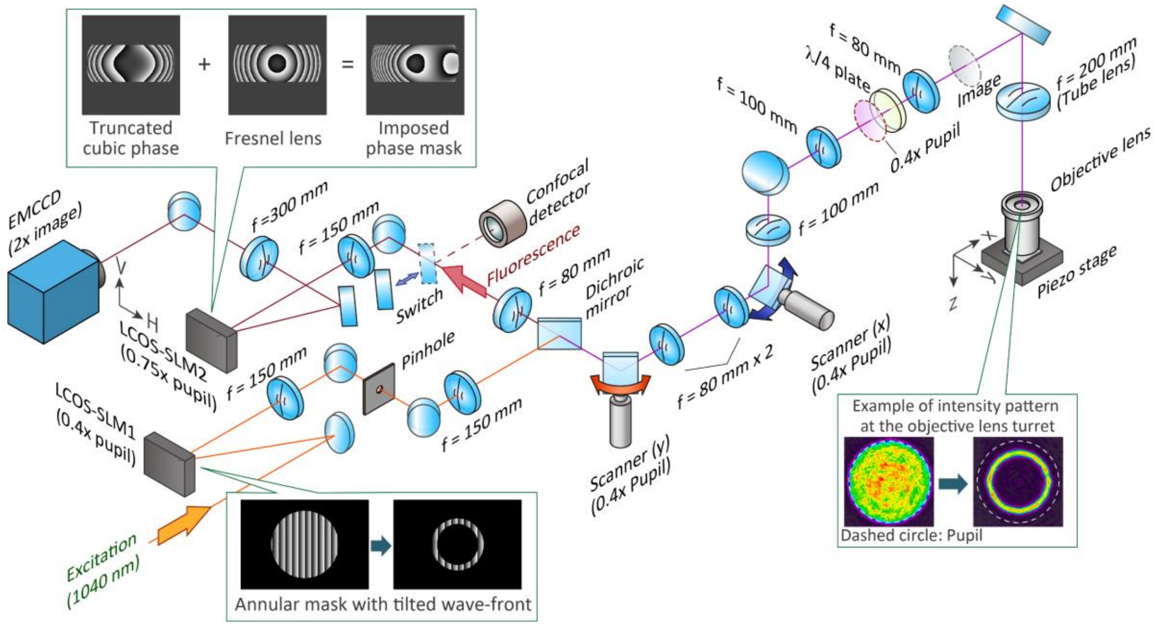


**Supplementary Figure S3.** Schematic diagram of the experimental setup. Two liquid crystal on silicon-SLMs (LCOS-SLM1 and LCOS-SLM2) were used to modulate the excitation beam and the fluorescent signal, respectively. Both LCOS-SLMs were located at the position in which the pupil plane of the objective lens was projected by relay optics with magnifications of 0.4 for excitation and 0.75 for detection. For conventional imaging, a flip mirror (denoted as “Switch” in the figure) was used to change the detection path to the confocal detector path.

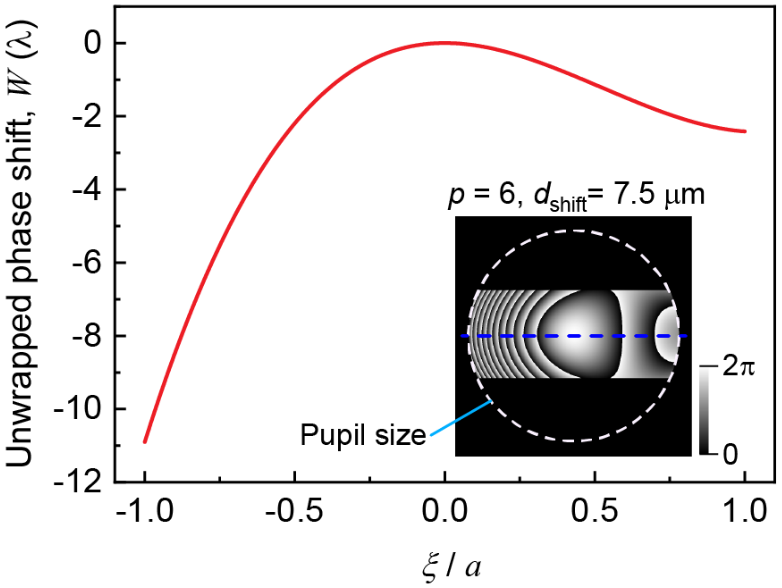


**Supplementary Figure S4.** Unwrapped phase shift for a phase modulation with $p$ = 6 and $d_{\mathrm{shift}}$ = 7.5 μm (shown in the inset) along the horizontal axis across the centre of the pupil plane (indicated by a blue dashed line in the inset). The steepest phase variation occurs at $\xi = -a$ , where its derivative is measured to be ~6 ($\lambda$/mm, where $\lambda$ = 560 nm). Near this point, the phase variation of 1$\lambda$ arises with 0.17 mm, corresponding to 16 pixels in our SLM.

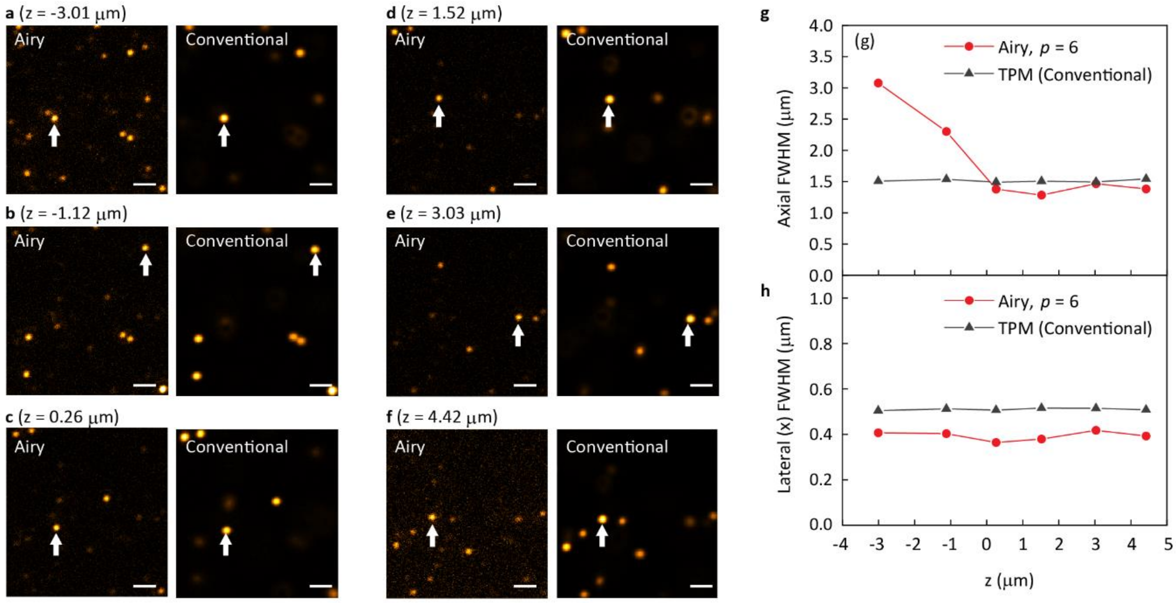


**Supplementary Figure S5.** Measured spatial resolution for PSFs with Airy beam conversion. (**a**–**f**) Reconstructed *xy* images of 200-nm orange beads embedded in agarose gel acquired via Airy beam conversion with $p$ = 6 and $d_{\text{shift}}$ = 7.5 μm (left panel) and conventional two-photon excitation microscopy (right panel) for $z$ = –3.01, –1.12, 0.26, 1.52, 3.03 and 4.41 μm. The scale bar in each panel is 2 μm. (**g**, **h**) The evaluated size of isolated bead images, indicated by an arrow in each panel, in the axial direction (**g**) and lateral direction along the *x* axis (**h**) as a measure of the spatial resolution.
